# Current State and Problems of Radiation Risk Communication: Based on the Results of a 2012 Whole Village Survey

**DOI:** 10.1371/currents.dis.84670981063d27f0a7c41b959fca70ec

**Published:** 2017-02-24

**Authors:** Yujiro Kuroda

**Affiliations:** Department of Public Health, Fukushima Medical University, Fukushima City, Japan

## Abstract

**Purpose:**

The entire village of Iitate was contaminated by radioactive material from the Fukushima Daiichi Nuclear Power Plant; even today, the residents remain evacuated. For the villagers, risk communication is an important element of recovery and maintaining health. This analysis focuses on the problem of radiation, presents results from a questionnaire of villagers, and examines methods for future risk communication activities.

**Subjects and Methods:**

In May 2012, anonymous surveys were sent to 2914 heads of households whose addresses were registered in Iitate. Their understanding of radiation and information needs were extracted from the answers.

**Results and Discussion:**

There were 1755 valid responses (61.4%). In relation to understanding, the most frequent answer was “There are numerous opinions and I do not know which one is true” (72.2%), followed by “I definitely want opportunities to learn more about how radiation is created” (41.6%). Residents felt that they could not determine which of the available information was reliable. The 60s+ age group responded more than younger age groups that “I do not have much information and do not know much about it,” “I do not know much about it, so I want to learn more,” and “I definitely want opportunities to learn more about how radiation is created.” Among information needs, “publications” (50.2%) and “community associations” (45.9%) received many responses; residents want study groups to be held at places and through media that give them regular opportunities to connect with each other. Residents in their 20s and 30s preferred “publications,” while those in their 40s, 50s, and 60s+ were more likely to request “community associations” and “resident meetings.” In addition, we found gender differences in both understanding and information needs. These results indicate that radiation and health risk communication should be addressed in a way that aligns with residents’ needs by age and gender.

## Introduction

After the 11 March 2011 Great East Japan Earthquake and nuclear disaster, residents living in affected areas experienced great physical and psychological changes. Five years later, the number of “disaster-related deaths”, such as from health deterioration and overwork, had risen to 3472 people in 10 prefectures, including 2038 from Fukushima Prefecture as of 31 March 2016, and more than 1607 deaths directly caused by the tsunami or earthquake^1^. The consequences of the accident have been reported both at home and abroad^2^^,^^3^^,^^4^.

Iitate village that is subject to this study is located 30-50km northwest of the crippled nuclear power plant, and had a pre-disaster population around 6000. Some parts of the village received a heavier dose of radiation than other towns far closer to the nuclear power plant^5^. However, they were not immediately evacuated because many residents felt safe following a medical expert’s lecture on radiation exposure at the end of March and the government’s announcement that “levels of radiation would not immediately affect the human body”^6^. Yet there was also a great sense of mistrust toward statements from experts, as well as toward the disclosure of information and the response from government and the public administration^7^.

Mistrust inhibits risk communication^8^^,^^9^^,^^10^. Risk communication has been deemed a pillar of recovery measures in Iitate^11^. Since the earthquake, the term has been used in a variety of settings. In this paper, it is defined as “promoting the sharing and discussion of information regarding radioactive contamination in order to support informed decision making”^11^. Mothers, village representatives, doctors and experts in radiation protection, and public officials in Iitate established the Health Risk Communication Promotion Committee in October 2012. The committee conducts study groups with radiation protection experts and village public health nurses in temporary housing locations and elementary and middle schools. It also publishes a quarterly magazine about health and radiation^12^, and disseminates information about radiation, such as to the villagers who remain evacuated outside the prefecture.

Individual differences influence perceptions of anxiety and risk; these differences may depend on the way risk is conveyed^13^^,^^14^. Slovic identifies the perception of dread and knowledge of risks as elements of risk perception^15^. Sandman adds outrage^16^. Radiation cannot be seen or felt, and education in Japanese schools has neglected radiation. Thus, radiation risk communication in Japan faces two major problems:

(1) Laypeople have difficulty understanding radiation itself^17^^,^^18^.

(2) With different standpoints, people understand “radiation” differently^19^.

This study analyzed data from a questionnaire sent to all Iitate residents after the evacuation to determine what residents wanted in risk communication.

## Methods

**1. Survey Method**

An anonymous self-response survey was sent out by the Iitate local government by mail from 22 May to 1 June 2012 to 2914 heads of households (or main income earners) with addresses registered in Iitate. This study used some of the results of that survey.

**2. Survey Items**

The survey comprised 30 items on opinions regarding life after evacuation, contamination in the village, and returning to the village. This report focused on 2 items relevant to radiation. The analysis for this study targeted items relating to radiation risk communication and basic attributes (age group, gender, occupation).

Iitate’s risk communication committee identified two items relevant to radiation risk communication. The first concerned the self-evaluated level of understanding. In answer to the question “When we think of the village residents’ health and future, we believe that radiation study groups (on risk communication) are necessary. What is your level of understanding about radiation and radioactive materials?”, respondents could choose from the following:

- I do not have much information and do not know much about it(Q1-1).

- I do not know much about it, so I want to learn more(Q1-2).

- There are numerous opinions and I do not know which one is true(Q1-3).

- I definitely want opportunities to learn more about how radiation is created(Q1-4).

- I know about it, so I do not need a study group(Q1-5).

- Other.

The second item concerned the format of study groups. In answer to the question “In implementing radiation study groups, what type of location would make them easy to attend?”, respondents could choose from the following:

- I would like them to take place in schools, in preschools, and at daycare parent–teacher association events(Q2-1).

- I would like them to be implemented through community associations of administrative districts and at temporary housing locations(Q2-2).

- I would like them to happen together with health lessons(Q2-3).

- I would like them to happen together with resident meetings(Q2-4).

- I would like them to be given in lecture halls by experts(Q2-5).

- I am extremely concerned about radiation and would like the study group to happen as an individual consultation(Q2-6).

- I would like information to be published in the form of special features and announced in public newsletters, flyers, and newspapers(Q2-7).

**3. Analysis**

Responses to answers by gender (male and female) and by age group (20s–30s, 40s–50s, and 60s+) were compared by chi-squared test in SPSS v. 19.0 software.

**4. Ethical considerations**

This survey was conducted by the Iitate village government. Data was provided to author to analyze and has no personal information. The Ethics Committee of Fukushima Medical University granted its consent (Examination No. 2609).

## Results

**1. Subjects’ backgrounds**

There were 1788 responses (60.2% of 2914). Excluding answers from teenagers left 1755 for final analysis, of which 15.5% were in their 20s–30s, 37.5% in their 40s–50s, and 47.0% 60 or older (Table 1). Males comprised 70.5% of the total and females comprised 29.5%. The most common occupation before evacuation was agriculture (31.9%), followed by working at a company (29.8%).

**Figure:**
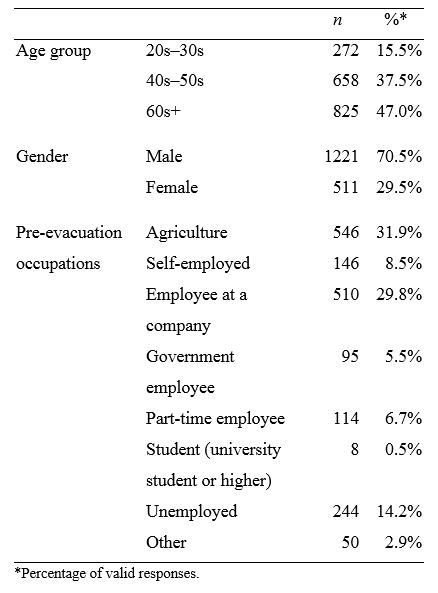
**Table 1**. Characteristics of subjects (n = 1755).

**2. Differences in level of understanding by age group and gender (Table 2)**

In relation to level of understanding, the answer “There are numerous opinions and I do not know which one is true” was chosen the most (72.2%), and “I know about it, so I do not need a study group” was chosen the least (4.4%; Table 2). Males were significantly more likely than females to respond “I do not know much about it, so I want to learn more” (χ2 = 12.89, P < 0.01), “I definitely want opportunities to learn more about how radiation is created” (χ2 = 13.25, P < 0.01), and “I know about it, so I do not need a study group” (χ2 = 6.09, P = 0.012). Older people more frequently responded with “I do not have much information and do not know much about it” (χ2 = 98.82, P < 0.01), “I do not know much about it, so I want to learn more” (χ2 = 64.72, P < 0.01), and “I definitely want opportunities to learn more about how radiation is created” (χ2 = 45.21, P < 0.01). People in their 40-50s gave the highest response to “There are numerous opinions and I do not know which one is true” (χ2 = 13.77, P < 0.01).

**Figure:**
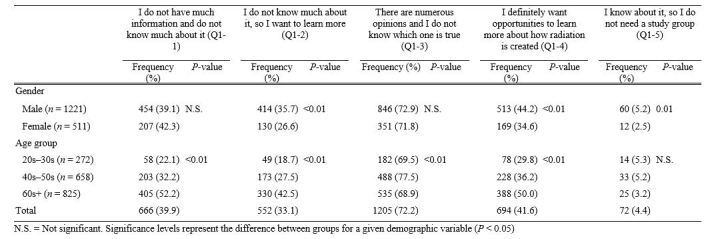
**Table 2**. Level of understanding about radiation: age group and gender distribution.

**Figure:**
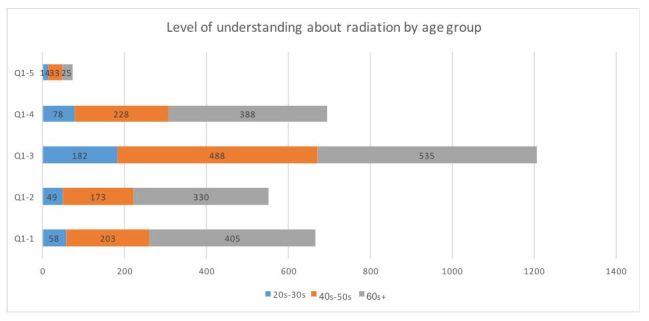
**Figure 1-1**. Level of understanding about radiation by age group (n=1755)

**Figure:**
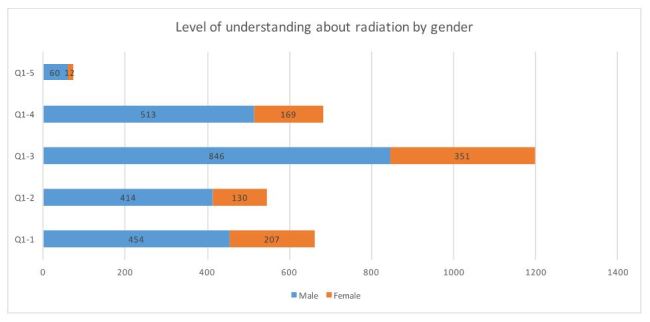
**Figure 1-2**. Level of understanding about radiation by gender (n=1733)

**3. Differences in study group requests age group and gender (Table 3)**

“Publications” received the highest response (50.2%) and individual consultations the lowest (13.4%; Table 3). Significantly more males than females requested “community associations” (χ2 = 20.21, P < 0.01), “resident meetings” (χ2 = 4.89, P = 0.02), and “lectures by experts” (χ2 = 9.65, P < 0.01). An age frequency bias (toward younger or older people) was found in “schools and preschools” (χ2 = 17.01, P < 0.01), “community associations” (χ2 = 125.46, P < 0.01), “health lessons” (χ2 = 22.16, P < 0.01), “resident meetings” (χ2 = 33.59, P < 0.01), “individual consultations” (χ2 = 19.25, P < 0.01), and “publications” (χ2 = 25.11, P < 0.01).

**Figure:**
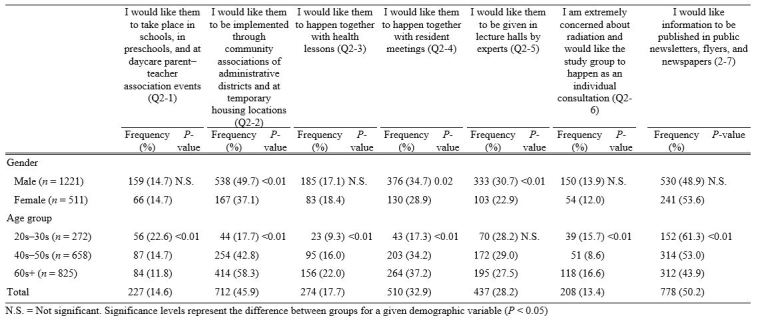
**Table 3**. Requests for study groups on radiation: age group and gender distribution.

**Figure:**
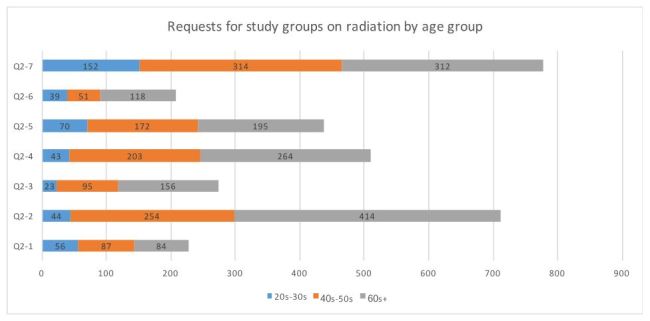
**Figure 2-1**. Requests for study groups on radiation by age groups (n=1755)

**Figure:**
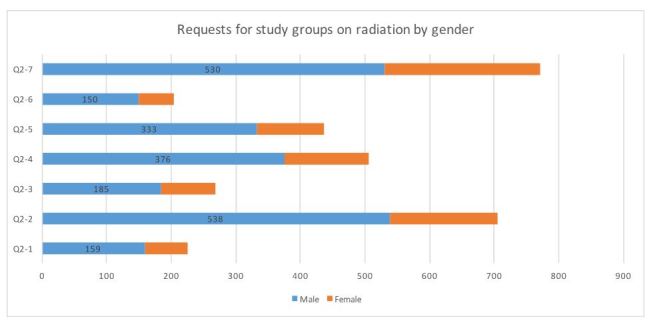
**Figure 2-2**. Requests for study groups on radiation by gender (n=1733)

## Discussion

The fact that a great number of residents chose “There are numerous opinions and I do not know which one is true” suggests that many of them feel overwhelmed by too much information on radiation. In addition, the fact that 40% chose “I do not have much information and I do not know much about it” and “I would definitely like opportunities to learn more about how radiation is created” indicates that residents did not receive information from authorities (such as the local village) that they trusted, and that it is necessary for both national and local government to reconsider the volume and quality of knowledge about radiation. The fact that over 50% of residents chose “publications” and over 45% chose “community associations” reveals a strong desire for media or places that allow people to connect regularly with each other, as well as a need to create mechanisms for risk communication that meet residents’ needs.

**Current state and problems of risk communication: analysis by age group**

Differences by age group were found in both the level of understanding about radiation and requests for study groups. The response “I do not have much information and I do not know much about it” was greater among those aged 60 and older than among those in their 20s–30s and 40s–50s. Responses indicating initiative—“I do not know much about it, so I want to learn more” and “I would definitely like opportunities to learn more about radiation how is created”—were also more common.

Although the village has been holding study groups on radiation for older villagers who live in temporary residences, it is possible that these villagers do not have enough opportunities and that they are not receiving information they want. So in addition to this current approach, it will be necessary to engage in dialogue with older villagers to learn which specialists and public health professionals can best meet their needs.

Unlike the older villagers, the younger villagers seem to strongly distrust the discourse on radiation. Figueroa suggests that immediately after a natural disaster, disseminating uncertain information hinders residents’ ability to make decisions, especially in young adults, and instead reinforces the sense of mistrust^20^. Covello and Sandman report that the two-way approach (in which “expert” and “lay” perspectives should inform each other) allays fears even among young people who are suspicious^21^. Furthermore, because losing the sense of control after an accident exacerbates mistrust^22^^,^^23^, a resident-participation approach that is an approach in which residents participate in shaping the decisions about that information to provide, how frequently to provide it, and the optimal medium for conveying the information might help residents regain that sense^24^.

While respondents in their 20s–30s showed a strong desire to implement study groups in schools or via publications, those in their 40s–50s and 60s+ preferred for them happen through community associations and resident meetings. In addition, the 60s+ group tended to want “health lessons”. These results show that many people want study groups to be held in places or via media that bring them into regular contact with others. In addition, 15.7% of the 20s–30s group and 16.6% of the 60s+ group were extremely worried about radiation and wanted individual consultations. This indicates the need for an individual approach that uses radiation experts or trained nurses in a medical setting.

The evacuation divided the community, and the residents’ lives changed completely. Although many people were living together in three-generation households, homes in which the young and the old lived separately increased. The number of senior citizens living alone also grew, and the total amount of households doubled from what it was before the evacuation^25^. As the community changes, we can see that the younger generation wants to obtain information at school events and through publications. Moreover, most senior citizens live in groups in temporary housing as opposed to rental housing provided by Fukushima prefectural government, and various events and study groups happen at group meeting centers. Since the need for study groups changes according to age, it is desirable to implement them in settings and in ways that make it easy for residents to participate.

**Current state and problems of risk communication: analysis by gender**

Gender differences were found in the level of understanding and in requests for study groups. There was no gender difference in the response “I do not have much information and I do not know much about it”; however, significantly more males than females chose the answers “I do not know much about it, so I want to learn more” and “I definitely want opportunities to learn more about how radiation is created.”

On the other hand, it is possible that the level of understanding is polarized, since a considerably larger proportion of males chose “I know about it, so I do not need a study group.” It has also been shown that men and women hold different concerns about what is considered a risk^26^^,^^27^: specifically, women have more concerns about life (e.g., effects on children, pregnant women, future generations), and men have greater economic concerns. An analysis of societal gender roles can improve understanding of these findings28 .

Men favored interpersonal approaches to study groups, such as through community associations and at resident meetings, while women tended to prefer the more indirect approach of publications. This, too, is related to societal gender roles: elderly men usually attend meetings at “places” such as community associations and resident meetings while elderly women prefer a more informal setting. In addition, 8.6% to 16.6% reported being “extremely concerned” about radiation. Detailed implementation system that allows a small number of residents to have two-way communication with experts, with whom they can share their health concerns about radiation^24^^,^^29^.

**Limitations and prospects**

This study used a survey targeting all residents of Iitate village, and presents valuable data on residents’ understanding of radiation and the methods of risk communication that they wanted. However, it has several limitations. Since the validity and reliability of the survey items have not been confirmed, it is necessary to take care in interpreting and generalizing the results. In addition, the survey could not examine the kind of risk communication content needed. The survey targeted the entire population of the village, not a sample, and was conducted as village policy; the question of how to interpret a failure to respond remains.

For future consideration, it is necessary to clarify the content that residents need, and to measure results on a scale whose reliability and validity have been verified. Since a failure to respond could be viewed as criticism of village policy, the potential engagement of a third party (such as a university or non-profit organization) needs to be examined. Furthermore, since the survey results were reported a year after the evacuation, it is possible that residents’ perceptions of radiation have now shifted. Beside conducting the survey again, it will be necessary to explore timely ways of communicating risk.

## Corresponding Author

Yujiro Kuroda, PhD (kuroday@fmu.ac.jp)

## Data Availability Statement

Data is available upon request from the author Yujiro Kuroda (kuroday@fmu.ac.jp)

## Competing Interest Statement

The authors have declared that no competing interests exist.
